# Comprehensive transcriptomic analysis of age-, dark-, and salt-induced senescence reveals underlying mechanisms and key regulators of leaf senescence in *Zoysia japonica*

**DOI:** 10.3389/fpls.2023.1170808

**Published:** 2023-05-30

**Authors:** Lanshuo Wang, Phan Phuong Thao Doan, Nguyen Nguyen Chuong, Hyo-Yeon Lee, Jin Hee Kim, Jeongsik Kim

**Affiliations:** ^1^ Interdisciplinary Graduate Program in Advanced Convergence Technology & Science, Jeju National University, Jeju, Republic of Korea; ^2^ Subtropical Horticulture Research Institute, Jeju National University, Jeju, Republic of Korea; ^3^ Department of Biotechnology, Jeju National University, Jeju, Republic of Korea; ^4^ Faculty of Science Education, Jeju National University, Jeju, Republic of Korea

**Keywords:** leaf senescence, transcription factor, transcriptome analysis, RNA-Seq, age, dark, salt, Zoysiagrass

## Abstract

The lawn grass *Zoysia japonica* is widely cultivated for its ornamental and recreational value. However, its green period is subject to shortening, which significantly decreases the economic value of *Z. japonica*, especially for large cultivations. Leaf senescence is a crucial biological and developmental process that significantly influences the lifespan of plants. Moreover, manipulation of this process can improve the economic value of *Z. japonica* by extending its greening period. In this study, we conducted a comparative transcriptomic analysis using high-throughput RNA sequencing (RNA-seq) to investigate early senescence responses triggered by age, dark, and salt. Gene set enrichment analysis results indicated that while distinct biological processes were involved in each type of senescence response, common processes were also enriched across all senescence responses. The identification and validation of differentially expressed genes (DEGs) *via* RNA-seq and quantitative real-time PCR provided up- and down-regulated senescence markers for each senescence and putative senescence regulators that trigger common senescence pathways. Our findings revealed that the NAC, WRKY, bHLH, and ARF transcription factor (TF) groups are major senescence-associated TF families that may be required for the transcriptional regulation of DEGs during leaf senescence. In addition, we experimentally validated the senescence regulatory function of seven TFs including *ZjNAP, ZjWRKY75, ZjARF2, ZjNAC1, ZjNAC083, ZjARF1*, and *ZjPIL5* using a protoplast-based senescence assay. This study provides new insight into the molecular mechanisms underlying *Z. japonica* leaf senescence and identifies potential genetic resources for enhancing its economic value by prolonging its green period.

## Introduction

The genus *Zoysia* Willd., belonging to the tribe *Zoysieae* and subfamily *Chloridoideae* (Poaceae), is found in temperate Northeast Asia, including Korea and Japan, as well as tropical China and Southeast Asia. *Zoysia japonica*, also known as Korean or Japanese lawngrass, is a popular warm-season C4 turf species because it requires little maintenance and can withstand environmental stresses such as heat, drought, and traffic (Teng et al., 2016a). However, the fact that *Z. japonica* has a relatively short green period compared to other cool season turfgrasses limits its widespread use. Therefore, turf breeders have attempted to develop *Z. japonica* cultivars with longer and healthier green periods (Teng et al., 2016a; Loch, 2017).

Leaf senescence is an evolutionally acquired process that occurs during the final stage of leaf development as an energy utilization strategy (Mahmood et al., 2022). Leaves are photosynthetic organs that produce chemical energy and building units for macromolecules and accumulate cellular materials during growth periods. During leaf senescence, chloroplasts begin to degrade and the macromolecules they contain, including lipids, proteins, and nucleic acids, are disassembled and transferred to growing organs such as new shoots, young leaves, seeds, or flowers. (Guo et al., 2021). Premature and delayed senescence can negatively affect plant quality and yield by reducing the accumulation and remobilization periods, respectively, underscoring the importance of initiating senescence at an appropriate time for maximizing progeny succession. Thus, leaf senescence is a vital life history strategy with significant biological consequences.

The onset of leaf senescence is determined by the coordinated actions of senescence regulatory genes, which are themselves controlled by chromatin, transcriptional, post-transcriptional, translational, and post-translational regulation. During leaf senescence, the expression of thousands of senescence-associated genes (SAGs) involved in various molecular and physiological processes are primarily regulated at the transcriptional level (Kim et al., 2014). The identification of multiple transcription factors (TFs) involved in leaf senescence regulation emphasizes the significance of TF-mediated transcriptional control. The WRKY, NAC (NAM, ATAF1/2, and CUC2), bHLH (basic helix-loop-helix), and MYB (myeloblastosis-related) TF families have all been identified as being involved in the regulation of the onset and/or progression of leaf senescence in plants (Miao et al., 2004; Kim et al., 2009; Jaradat et al., 2013; Qi et al., 2015; Kim et al., 2016a). In addition to regulating the transcription of downstream SAGs, regulatory TFs are interconnected with multiple crosstalk pathways and therefore have been shown to respond to various internal and external senescence-triggering signals including aging, phytohormones, darkness, salinity, heat, and disease (Buchanan-Wollaston et al., 2005; Breeze et al., 2011; Allu et al., 2014). Further research is needed to understand the transcriptional regulation of SAGs under different senescence-inducing conditions to identify common and unique aspects of their regulation (Zhang et al., 2014). Comparative transcriptomic approaches may be used to identify potential senescence regulatory genes that can delay the onset of senescence and thereby extend the green period of *Z. japonica*.

Over the past three decades, significant advances have been made in our understanding of the molecular mechanisms underlying leaf senescence in Arabidopsis (Kim et al., 2018b). The practical value of senescence programs has also created interest in molecular and genetic studies of leaf senescence in agricultural crops (Guo and Gan, 2014), including rice (Lee et al., 2001; Lee and Masclaux-Daubresse, 2021), tobacco (Uzelac et al., 2016), wheat (Uauy et al., 2006), maize (Zhang et al., 2014), and cotton (Kong et al., 2013). However, to date few studies have addressed leaf senescence in *Z. japonica*, despite the potential economic benefits of studying senescence-related phenotypes—such as visual greenness, nutritional levels, and biomass accumulation—in turfgrass (Teng et al., 2016a). Overexpression of *ZjSGR, ZjPPH*, and *ZjNOL* genes involved in chlorophyll degradation has been found to result in rapid leaf yellowing and/or early senescence phenotypes in Arabidopsis (Teng et al., 2016b; Teng et al., 2021; Guan et al., 2022a). In addition, approximately 200 SAG genes were identified as potential markers of senescence *via* subtractive hybridization of dark-induced leaf senescence in *Z. japonica* (Cheng et al., 2009). However, the genetic resources available for studying senescence in *Z. japonica* are limited (Wei et al., 2015; Tanaka et al., 2016), and the mechanisms underlying senescence in this species are not fully understood.

In this study, we used RNA sequencing (RNA-seq) analysis to compare age-, dark-, and salt-induced leaf senescence in order to understand the molecular basis of senescence in *Z. japonica* and to identify key regulatory genes. Differentially expressed genes (DEGs) were identified in each senescence condition and a group of DEGs that responded specifically to each type of senescence treatment were validated as molecular markers specific to individual senescence conditions. Functional categorization of DEGs revealed both unique and common biological processes associated with each senescence condition. In addition, using a protoplast-mediated transient expression system, seven TF genes that were responsive to all senescence conditions were identified as potential regulators of all senescence conditions. This study therefore provides a molecular foundation for understanding *Z. japonica* leaf senescence and identifies genes that may be useful for future genetic modification of *Z. japonica* to extend its leaf greening period.

## Materials and methods

### Plant material and growth conditions

*Z. japonica* Steud. cv Duckchang, a wide-leaf variety, was used for all experiments (Duckchang Agri-Business Co., South Korea). Grasses were vegetatively planted *via* rhizomes in pots and grown in a greenhouse under supplementary white LED lighting (at a neutral white light temperature of 4,000K), which was provided on cloudy or rainy days. The temperature in the greenhouse was maintained at 30°C–35°C during the day and at 20°C–25°C at night. For senescence assays, the fourth leaves emerging from sprouts were cut into three pieces, of which the middle part was used for all assays. Leaf age was measured in days after leaf emergence (DAE).

### Plasmid construction

Pfu-X DNA polymerase (Solgent, South Korea) was used to amplify candidate TFs and promoters using *Z. japonica* leaf cDNA and genomic DNA as templates, respectively, using the corresponding primer sets (**Supplementary Table 1**). PCR products were then subcloned into pCR-CCD-F entry vectors after being digested with the appropriate restriction enzymes. The GATEWAY cloning technology (Invitrogen) was used to generate plasmid constructs for the effectors and reporters used in the protoplast-mediated transient expression assay. For overexpression effectors in protoplasts, the Gateway version of pCsVMV-eGFP-N-999 was used to recombine with corresponding entry clones to generate the following effector plasmids: ZjNAP-, ZjWRKY75-, ZjNAC1-, ZjAZF2-, ZjNAC083-, ZjARF1-, ZjPIL5-, and ZjHB2-pCsVMV-eGFP-N-999 (Kim and Somers, 2010). For protoplast reporter plasmids, including ZjSGR- and ZjPCAP-LUC, all promoter-LUC final constructs were established by LR recombination using the corresponding entry clone and the Gateway version of the pOmegaLUC_SK vector.

### Age-, dark-, and salt-induced senescence assays

The middle parts of leaves were collected at DAE 21 and used for dark- or salt-induced senescence assays or as a mature green sample to act as a control for the age-induced senescence assay (Hayakawa et al., 2006). For the dark-induced leaf senescence assay, leaf samples were first floated upside down on 3 mM MES buffer (pH 5.7) in 12-well plates, which were wrapped in aluminum foil. Samples were then incubated at 25°C for the indicated number of days and were collected to analyze 4–5 h after light-on. For salt-induced leaf senescence assays, leaf samples were prepared as described for the dark assay, except that they were incubated in 3 mM MES buffer supplemented with 150 mM NaCl in 16 h light/8 h dark conditions. For age-induced senescence assays, the middle region of the fourth leaves, collected at the specified age and at DAE 42 for RNA-seq analysis, was collected 4–5 h after light-on. All collected leaf samples were either used to quantify chlorophyll content and photochemical efficiency or stored at −80°C for further gene expression or transcriptome analysis. For each assay, eight to twelve leaves were used to determine chlorophyll content using a CCM-300 chlorophyll content meter (Opti-Sciences, USA) and photochemical efficiency using a Pocket PEA chlorophyll fluorimeter (Hansatech Instruments, UK). At least nine leaves were collected for each condition for gene expression or transcriptome analysis, and three biologically independent samples were obtained by vegetative planting of independent rhizomes.

### RNA sequencing and bioinformatic analyses

Total RNA was extracted from all collected samples using WelPrep™ Total RNA Isolation Reagent (Welgene, South Korea). We then determined the integrity of total RNA using RNA Pico 6000 chip kits for the Agilent Technologies 2100 Bioanalyzer. Samples with RNA integrity values ≥ 7.0 were retained for further analyses. RNA-seq was performed by Macrogen (South Korea). A TruSeq Stranded mRNA LT Sample Prep Kit for Illumina sequencing was used to create mRNA-seq libraries; all procedures followed the manufacturer’s instructions. Three independent sets of samples were used as biological replicates, and the generated library was subjected to pair-end 101-nt sequencing on an Illumina Novaseq 6000 sequencer. The quality of raw and trimmed sequences was checked using FastQC version 0.11.7. Raw sequence reads were preprocessed by removing adaptor sequences and by trimming low-quality ends using Trimmomatic version 0.38 (Bolger et al., 2014). Preprocessed reads were then aligned to the *Z. japonica* ssp. Nagirizaki genome sequences (Tanaka et al., 2016) using HISAT2 version 2.1.0 with all parameters set to the default settings (Kim et al., 2019). We created a new *Z. japonica* genome annotation file using StringTie Merge (Pertea et al., 2015) with a set of transcripts with minimum FPKM level of 0 and 15 resultant BAM files (**Supplementary File 1** and **Dataset 1**). Next, all reads in annotated genes were assembled using StringTie version 2.1.3b (Pertea et al., 2015), using the newly created *Z. japonica* genome annotation files. Protein and cDNA sequence of the annotated genes with new gene identification (ID) numbers were obtained from TransDecoder version 5.5.0 (Haas et al., 2013) and gffread version 0.12.7 (Trapnell et al., 2010), respectively and their gene structure was matched and compared with old *Z. japonica* gene ID (**Supplementary Dataset 1**). The abundance of gene expression was determined by using quantile normalization of fragments per kilobase million (FPKM) values after adding 1 to each FPKM value to avoid negative infinity (**Supplementary Dataset 2**). Genes with more than one count were statistically analyzed by pairwise comparison. Principal component analysis (PCA) was then performed by PCAGO for each transcriptome dataset using the expression profiles of 35,985 genes (Gerst and Hölzer, 2018).

Next, we identified DEGs for each senescence-induced condition using the DESeq-R package. This analysis was based on regularized log (rlog)-transformed values of FPKM and Relative Log Expression normalization (Love et al., 2014). DEGs were selected using a |log_2_ fold-change (FC)| cut-off of ≥ 1 and an adjusted p-value cut-off of < 0.05, as determined by the nbinomWaldTest function as implemented in DESeq-R. Functional analysis of all DEGs was performed using MapMan (Thimm et al., 2004) and data were then visualized using PageMan (Usadel et al., 2009). A critical Z-score value of 1.96 (equivalent to a p-value = 0.05) was used to select enriched functional categories. Gene ontologies for *Z. japonica* were established *via* sequence homology-based gene categorization with Mercator used to predict the protein sequences of new *Z. japonica* gene IDs (Lohse et al., 2014). RNA-seq data have been deposited at the NCBI Sequence Read Archive (BioProject, PRJNA934408).

### Gene expression analysis *via* qRT-PCR

Frozen leaf tissues were homogenized using a Retsch Mixer Mill MM400 (Retsch GmbH, Haan, Germany) and total RNA was then isolated using WelPrep™ Total RNA Isolation Reagent (Welgene, South Korea). RNA was treated with DNase I (Ambion, USA) to remove genomic DNA contamination, and 0.75 μg of the resulting total RNA solution was reverse-transcribed into cDNA in a 10-μL reaction volume using an oligo (dT15) primer and ImProm-II™ reverse transcriptase (Promega). The generated cDNA was then diluted 12-fold and 3 μL of diluted cDNA was amplified using TOPreal™ qPCR 2X PreMIX (Enzynomics, Korea) and a CFX96 real-time qPCR detection system (Bio-rad). Gene-specific primers (**Supplementary Table 2**) were used to quantify the corresponding transcript levels. The PCR program included denaturation at 95°C for 1 min, followed by 15 sec at 95°C and 34 sec at 60°C for 40 cycles. The comparative CT approach (Livak and Schmittgen, 2001) was used to quantify fold changes in gene expression, with actin (i.e., *ZjACT*) serving as the reference gene. Relative gene expression for the kinetic analysis was calculated by normalizing the gene expression levels of each condition with the maximal levels found in control samples. At least two biological replicates were included for each condition.

### Transient expression in Arabidopsis protoplasts

Protoplast isolation and DNA transfection were performed as previously described (Doan et al., 2022). Briefly, ten to fifteen leaves of three- to four-week-old Col-0 plants grown in 16 h light:8 h dark photocycles were treated with 70% ethanol for 30 sec and rinsed twice with sterile water. The leaves were then scratched lightly with sandpaper and incubated for 2.5 h at room temperature in 10 mL of cell wall digestion enzyme solution (1% Cellulase R10, 0.5% Macerozyme R10 [Yakult Honsha, Japan], 400 mM mannitol, 20 mM KCl, 10 mM CaCl_2_, 20 mM MES-KOH [pH 5.7], and 0.1% BSA [Sigma A6793]). The protoplast solutions were then filtered using 100 μm nylon mesh and centrifuged at 100 x g for 5 minutes in a round-bottom culture tube. Next, the precipitated protoplasts were washed with 1 mL of W5 solution (154 mM NaCl, 125 mM CaCl_2_, 5 mM KCl, 1.5 mM MES-KOH [pH 5.7], and 5 mM glucose) and placed on ice for 30 min. The protoplasts were then harvested and resuspended in MMG solution (400 mM mannitol, 15 mM MgCl_2_, and 4 mM MES-KOH [pH 5.7]), at a final cell concentration of 2 × 10^5^ mL^−1^. In addition, plasmid mixtures—including 25 μL effector, 5 μL reporter, and 0.2 μL of an internal control (35S-RLUC)—were added to 200 μL of the protoplast MMG solution. The plasmid DNAs used for transfection were prepared *via* CsCl gradient ultracentrifugation (Bio-Health Materials Core-Facility, Jeju National University, South Korea), and their final DNA concentration was adjusted to 2 μg μL^−1^ per 4 kb DNA. Protoplasts containing plasmid DNA were transfected by adding 230 μL (1 vol.) of polyethylene glycol (PEG) solution [40% PEG-4000, 200 mM mannitol, and 100 mM Ca(NO_3_)_2_] and incubating this mixture for 8 to 15 min at room temperature. The protoplast-DNA-PEG mixture was then diluted with 920 μL (2 vol.) of W5 solution. After 1 min centrifugation at 100 x g, transfected protoplasts were resuspended in 700 μL of W5 solution containing 5% fetal bovine serum (Sigma F4135) and 50 μg ml^−1^ ampicillin. Next, 300 μL aliquots of transfected protoplasts were placed in each well of a white 96-well microplate containing 3 μL of LUC substrate (5 mM luciferin [Goldbio LUCK-250, Netherlands]) or 3 μL of RLUC substrate (10 μM Coelenterazine-native, Sigma C2230). The microplate was then sealed with a transparent plastic cap and incubated on a GloMax 96 microplate luminometer (Promega) at 22°C in the dark. Luminescence levels were measured every 30 min for three days. Relative LUC reporter activity was determined by normalizing the firefly luciferase bioluminescent level of each data set to the highest RLUC level of all measurements in the same experiment.

## Results

### Preparation of leaf samples for comparative transcriptomic analysis of age-, dark-, and salt-induced senescence in *Z. japonica*

**Figure 1:**
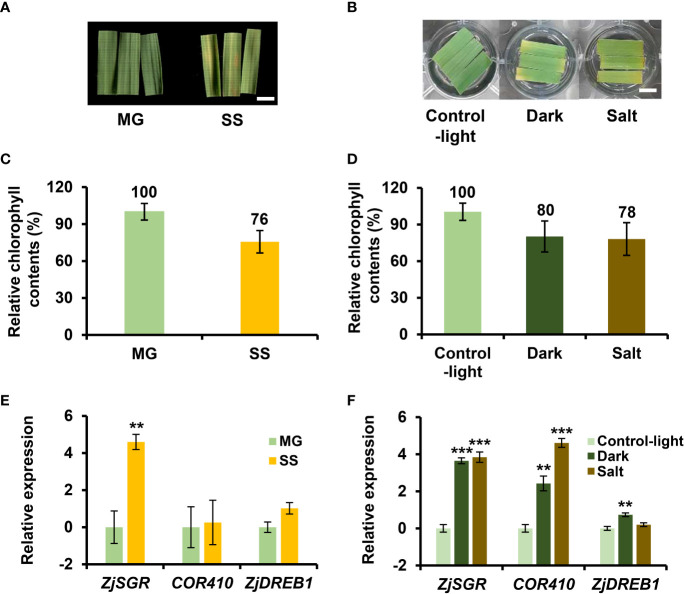
Preparation of leaf samples for comparative transcriptome analysis of age-, dark-, and salt-induced senescence in *Z. japonica*. **(A, C, E)** For age-induced senescence, leaf fragments at days after emergence (DAE) 21 and 42 were harvested as mature green (MG; control for AGE) and senescence stage (SS; senescence sample for AGE) samples, respectively **(B, D, F)** For dark- and salt-induced senescence, leaf fragments at DAE 21 were placed in 3 mM MES buffer under light (Control-light; control for DARK and SALT), wrapped in aluminum foil (Dark), or in the MES buffer containing 150 mM NaCl (Salt) and leaf fragments were then harvested at DAT 4. AGE, DARK, and SALT indicate that senescence responses were induced by age, dark, and salt relative to each control, respectively, while MG, SS, Dark, and Salt indicate each sample. The fourth leaves emerging from a bud were cut at the indicated DAE into three pieces and the middle part was used for senescence assays. Shown are representative pictures of the leaf fragments for the MG and SS stages in age-induced senescence **(A)** and in dark- and salt-induced senescence and Control-light **(B)**. Leaf fragments of SS samples **(C)** and those of Dark and Salt samples **(D)** contained 75%–80% of the chlorophyll content of their respective controls, indicating early senescence. Bar = 1 cm. Data represent mean ± standard error of the mean (SE; n = 6). Shown are the expression levels of senescence- and stress-related genes in MG and SS samples **(E)**, and in Control-light, Dark, and Salt **(F)** samples. Gene expression was determined by qRT-PCR and normalized against *ZjACT* expression. qRT-PCR data represent mean ± SE (n = 3) and are presented as the log_2_(FC) calculated between each condition and their respective controls. Statistical analyses were performed using one-way ANOVA test (^**^p < 0.01 and ^***^p < 0.001).

Leaf senescence is an age-dependent process but is also influenced by various senescence-inducing factors including dark and salt (Bengoa Luoni et al., 2019). Dark and salt have been widely used to mimic starvation and abiotic stress in senescence assays (Allu et al., 2014; Lee and Masclaux-Daubresse, 2021). However, although the biochemical and physiological responses to different senescence conditions were similar, the transcriptional responses to senescence are especially diverse at the initial senescence stage (Lim et al., 2007; Woo et al., 2019). To identify unique and common molecular mechanisms underlying various senescence responses in *Z. japonica*, we conducted a comparative transcriptomic analysis of age-, dark-, and salt-induced senescence. We monitored temporal changes in chlorophyll content, a typical indicator of senescence, during age-, dark-, and salt-induced senescence (**Figures 1A, B**). Leaves with nearly 80% of their initial chlorophyll levels in each senescence condition were classified as being in the early senescence stage. With respect to age-induced senescence, we measured the chlorophyll content of fourth leaves every four-day interval during leaf aging. Since chlorophyll levels in leaves at DAE 21 reached their maximum levels as they aged, DAE 21 leaves were considered to be at the mature green (MG) stage, which was used as a reference point for age-induced senescence as well as a starting material for dark- or salt-induced senescence assays (see Methods). Chlorophyll levels in leaves gradually decreased with aging and DAE 42 leaves were found to contain 76% to 80% of the chlorophyll levels of DAE 21 leaves (referred to as MG) (**Figure 1C**). DAE 42 (referred to as SS) and DAE 21 leaves were selected as the age-induced senescence and control samples for transcriptome analysis, respectively.

For dark- and salt-induced senescence, DAE 21 leaves were incubated in MES buffer in the dark and in MES buffer containing 150 mM NaCl under light, respectively. We monitored their chlorophyll levels and compared these to the levels of leaves kept in MES buffer under light over the course of treatment. At four days after treatment (DAT), we found that the dark- and salt-induced senescence conditions both contained 80% of the chlorophyll levels of DAT 4 leaves in MES buffer under light. These leaves were selected as dark- and salt-induced senescence samples (hereafter referred to as “Dark” and “Salt”, respectively). Both these samples and control samples (hereafter referred to as “Control-light”) were then submitted for transcriptome analysis (**Figure 1D**). In addition, the expression levels of *ZjSGR*, *COR410*, and *ZjDREB1*, age- and stress-associated marker genes in *Z. japonica* (Kidokoro et al., 2015; Teng et al., 2016a; Li et al., 2019) were examined alongside control leaves by qRT-PCR to validate the senescence-related responses. We found that the transcription levels of *ZjSGR*, *COR410*, and *ZjDREB1* increased preferentially in the SS, Salt, and Dark samples relative to their respective controls (i.e., MG and Control-light; **Figures 1E, F**). This indicated that the samples used for transcriptome analysis showed differential molecular responses that may reflect differences among senescence conditions, as well as that differential molecular responses are involved in age-, dark-, and salt-induced conditions, as has been previously reported (van der Graaff et al., 2006; Zhang et al., 2014).

### Transcriptomic comparison of age-, dark-, and salt-induced senescence

To investigate the molecular basis of age-, dark-, and salt-induced leaf senescence in *Z. japonica*, we conducted mRNA sequencing analysis on five biological sample sets (i.e., MG, SS, Control-light, Dark, and Salt). Each condition was analyzed using three biological replicates. The resulting dataset was used to create three comparison sets to examine age-induced senescence (i.e., MG vs. SS; for “AGE”), dark-induced senescence (i.e., Control-light vs. Dark; for “DARK”), and salt-induced senescence (i.e., Control-light vs. Salt; for “SALT”). Library construction and sequencing were performed on a total of 15 samples, which generated a total of 124.1 Gb of high-quality clean data.

**Figure 2:**
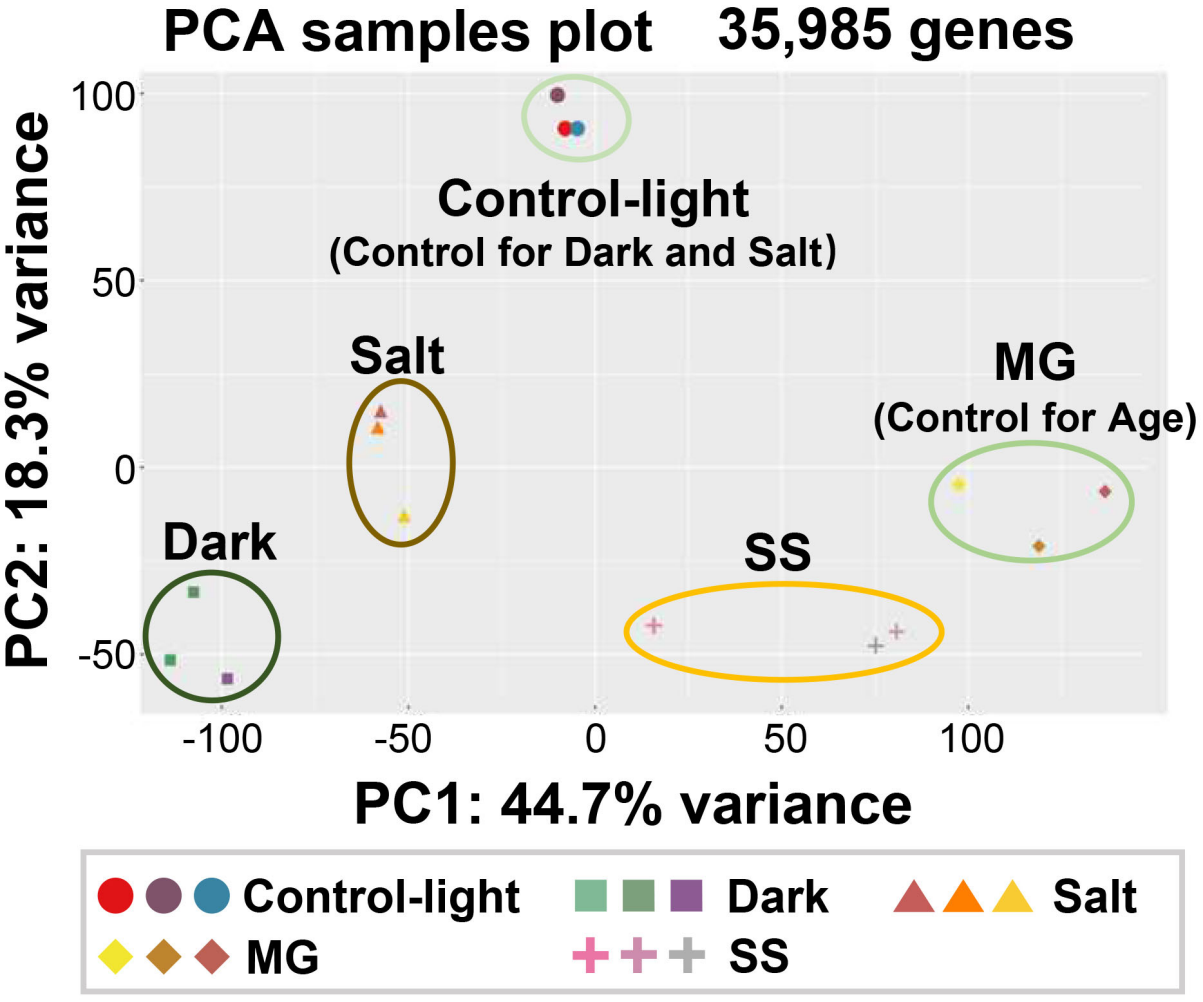
Principal component analysis of transcriptomic responses of *Z. japonica* leaves subjected to different senescence-inducing conditions. Transcriptome samples were prepared as indicated in **Figure 1**. Three biological samples per each condition were used. RNA-seq was performed using total RNA extracted from each sample. Principal component analysis (PCA) was performed using the expression profiles of 35,985 genes using PCAGO. PC1 and PC2 explained 44.7% and 18.5% of the total variance, respectively.

The GC content of the raw reads ranged from 51.8% to 53.4%, with Q30 percentages exceeding 96.5% (**Supplementary Table 3**). Between 73.1% and 95.3% of the total clean reads from each sample were mapped to the *Z. japonica* reference genome (**Supplementary Table 4**). However, the average matching rate of transcripts was approximately 75.2%, which is relatively low (Lin et al., 2020). Visual examination of the structural annotation data of reads mapped to the *Z. japonica* genome revealed that this low mapping rate was due to inaccurate transcript annotation. Therefore, we rebuilt the genome structural annotation with a new gene identification (ID) system based on our RNA-seq data. We then provided a comparison table between the new and old gene IDs in the *Z. japonica* genome (**Supplementary Dataset 2**). Using this new genome annotation, the mapping ratio of reads to transcripts increased to 88.8%−95.4% (**Supplementary Table 4**). We then calculated gene expression levels as FPKM values and normalized them among the 15 samples. Among *Z. japonica* nuclear genes, 52.6% (68,383 genes) were expressed in the leaves under our experimental conditions. We further conducted PCA on the genes expressed in each condition to validate the quality of our samples (Dong et al., 2022b). This PCA revealed that the transcriptomic profiles of the five sample sets were well separated into five clusters. Moreover, each biological replicate belonged to the same cluster, indicating that our RNA-seq dataset was reliable in reflecting each senescence-induced condition (**Figure 2**).

### Functional characterization of transcriptomic responses for age-, dark-, and salt-induced senescence

**Figure 3:**
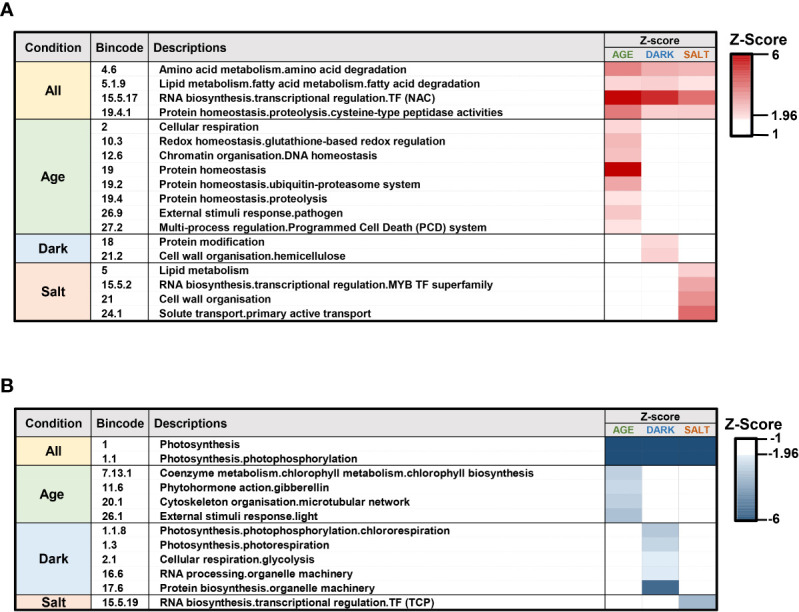
Gene set enrichment analysis for transcriptome profiles of various senescence-inducing conditions. Gene set enrichment analysis was performed using transcriptome profiles for each senescence condition using the PageMan modules as implemented by MapMan. The functional categories with significant increased **(A)** and decreased **(B)** expression profiles are shown for age-, dark-, and salt-induced senescence conditions. Gene ontologies for *Z. japonica* were established *via* sequence homology-based gene categorization with predicted protein sequences of new *Z. japonica* gene IDs.

To comprehend and dissect the biological processes occurring during senescence, we performed enrichment analysis of functional categories within defined gene sets using transcriptomic profiles containing the genes expressed for each senescence condition. We used PageMan to perform gene set enrichment analysis in functional categories assigned by MapMan. This analysis condensed 35,985 expressed genes into approximately 5,257 categories (**Supplementary Dataset 3**). Bincode and a threshold of |Z-Score| > 1.96 were then used to filter out categories that did not demonstrate significantly distinct changes in any conditions. Functional categories that are significantly up- or down-regulated relative to the rest of the array are shown (**Figure 3**). Our analysis showed that transcriptomic changes reflecting different senescence-inducing conditions have a significant impact on specific biological processes. Genes involved in the degradation of proteins, amino acids, and fatty acids, as well as NAC TF were among those found to be up-regulated while photosynthesis-related processes exhibited the most down-regulated gene enrichment (**Figure 3**). This analysis also highlighted the effect of specific senescence-inducing conditions on distinct processes. For example, cellular respiration, redox regulation, chromatin organization, and programmed cell death were the enriched biological processes in response to age-induced senescence, and within the protein degradation category, autophagy and ubiquitin-associated pathways were the major up-regulated categories. In contrast, genes related to gibberellin (GA) synthesis and cytoskeleton organization were significantly down-regulated. In response to dark-induced senescence, we found increased expression of genes involved in hemicellulose synthesis and protein modification, while genes involved in RNA processing, glycolysis, and protein biosynthesis were down-regulated. Finally, under salt-induced senescence conditions, we observed up-regulation in genes related to solute transports and changes in MYB and TCP TF families, with up- and down-regulation respectively. Overall, our transcriptomic analysis suggested that there exist both a comprehensive set of biological processes related to all senescence conditions as well as those that relate specifically to different types of senescence condition.

### Identification of DEGs

**Figure 4:**
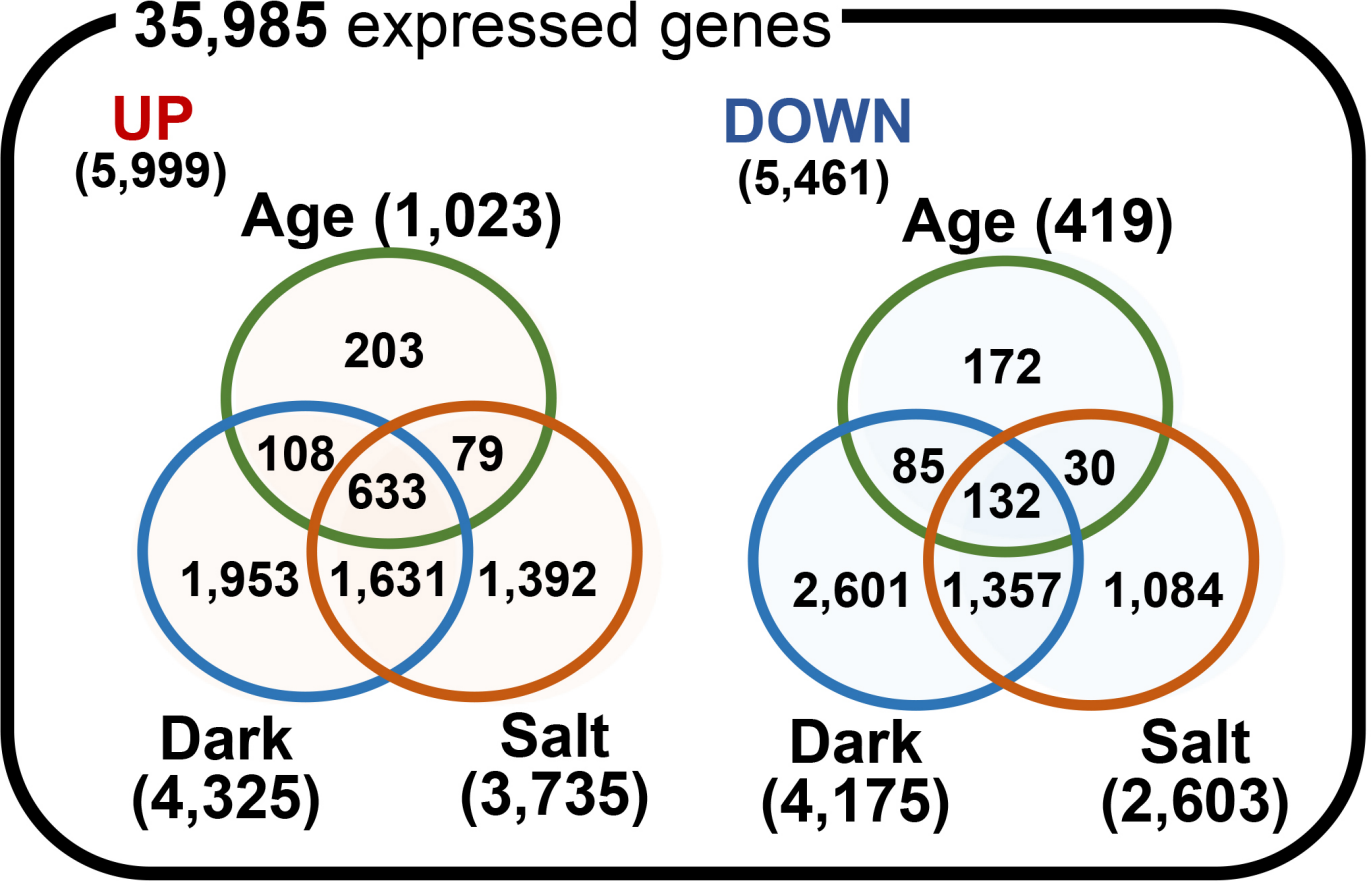
Comparison of differentially expressed genes for various senescence-inducing conditions. Venn diagrams depicting the number of up (UP)- and down (DOWN)-regulated genes for the age-, dark-, and salt-induced senescence conditions. Differentially expressed genes (DEGs) for each comparison pair were identified by a cut-off of |log_2_(FC)| ≥ 1 and a significance-adjusted p-value of <0.05, using the nbinomWaldTest as implemented in the DESeq2 R package.

We identified DEGs among the 35,985 expressed genes by performing pairwise comparisons of gene expression between each senesced and control condition. To do so, we used a selection threshold that included an adjusted p-value of < 0.05 and |log_2_(FC)| ≥ 1. A total of 1,442, 8,500, and 6,338 genes (representing 4.0%, 23.6%, and 17.6% of all 35,985 expressed genes, respectively; **Supplementary Dataset 4**) were identified as significant DEGs for the age-, dark-, and salt-induced senescence responses, respectively (**Figure 4**). These included 1,023, 4,325, and 3,735 up-regulated genes (representing 17.1%, 72.1%, and 62.3% of all 5,999 up-regulated genes [UP]) and 419, 4,175, and 2,603 down-regulated genes (representing 7.7%, 76.5%, and 47.7% of all 5,461 down-regulated genes [DOWN]) for the age-, dark-, and salt-induced senescence conditions, respectively. Furthermore, we also identified 203, 1,953, and 1,392 DEGs in the UP group and 172, 2,601, and 1,084 DEGs in the DOWN group that responded specifically to the age-, dark-, and salt-induced senescence responses, respectively. We also note that a higher number of DEGs were obtained for the conditions in which senescence was triggered by dark and salt than by age. This may be due to the longer period required for age-induced senescence, which may have therefore resulted in higher variability. Interestingly, we identified 633 UP (i.e., 1.76% of 35,985) and 132 DOWN (i.e., 0.367% of 35,985) DEGs that co-responded to all senescence-inducing conditions (referred to as cDEGs), and these cDEGs were highly enriched among DEGs, as they showed fold enrichments of 49.6 and 37.5 for UP and DOWN cDEGs, respectively. Taken together, these findings suggest that each senescence condition uses both a distinct and a shared set of genes during the senescence process, and that these DEGs/cDEGs may be involved in the occurrence and progression of leaf senescence in *Z. japonica*.

### Analysis of DEGs responding specifically to each senescence-inducing condition

**Figure 5:**
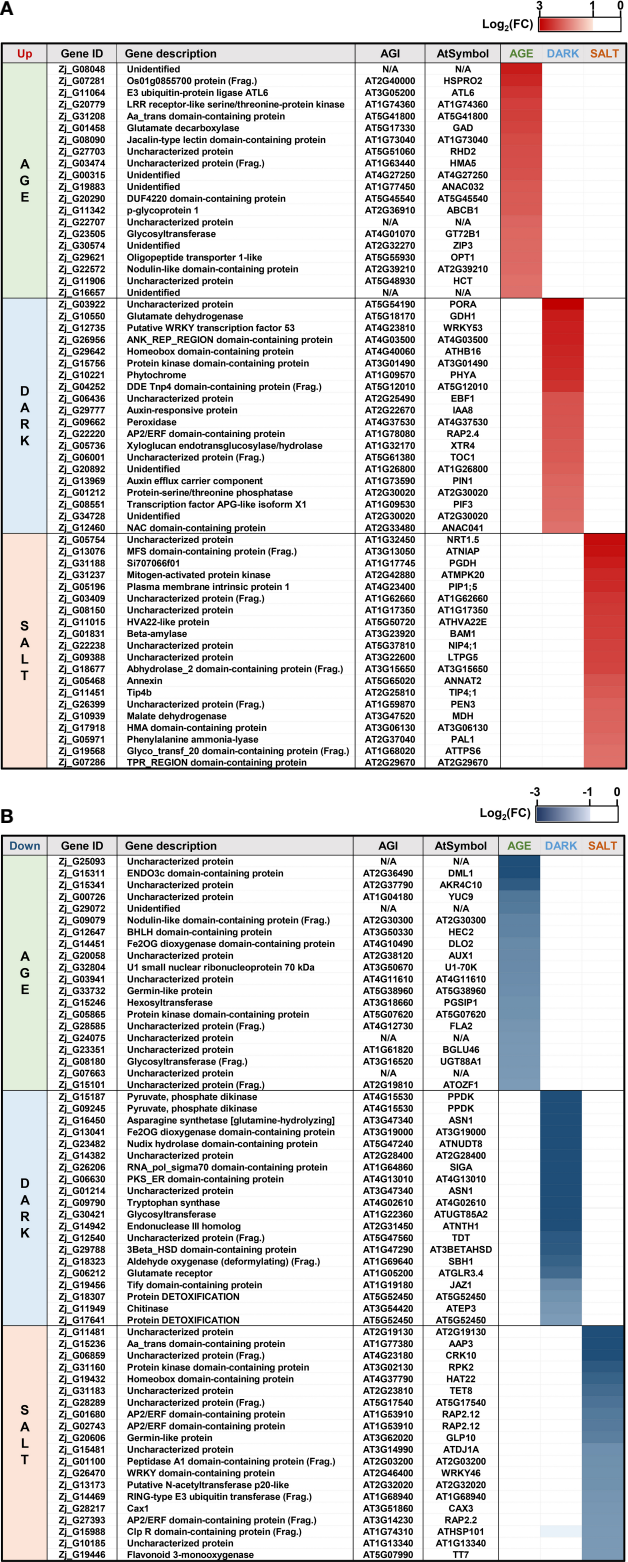
Specifically responsive genes for each senescence-inducing condition. Shown are the expression levels of DEGs specifically responding to the age-, dark-, and salt-induced senescence conditions. Heatmaps use color intensity to indicate the log_2_(FC) in DEG expression for each condition relative to their respective controls. Shown are heatmaps for the expression of up-regulated **(A)** and down-regulated **(B)** DEGs for each senescence condition. AGI and AtSymbol indicate the Arabidopsis gene index and gene symbol of a homologous Arabidopsis gene of DEGs, respectively.

**Figure 6:**
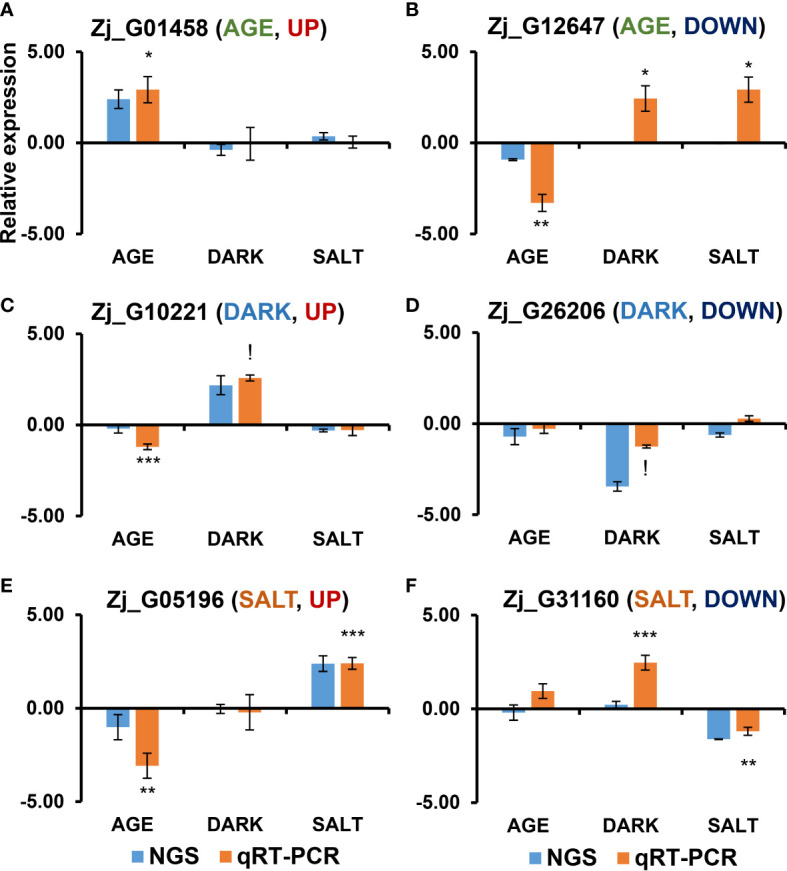
qRT-PCR-based validation of DEGs that were responsive in each senescence-inducing condition *via* RNA-seq analysis. Shown are the expression profiles of DEGs that were identified as up-regulated **(A, C, E)** and down-regulated **(B, D, F)** in each senescence condition in response to age **(A, B)**, dark **(C, D)**, and salt **(E, F)** *via* RNA-seq analysis. The samples used in this qRT-PCR validation were independently prepared from those used in the RNA-seq analyses. Data represent mean ± SE (n = 4) and are presented as the log_2_(FC) calculated between each condition and their respective controls. Statistical analyses were performed using one-way ANOVA test (*p < 0.05; **p < 0.01; ***p < 0.001; and ^!^p < 0.0001).

Diverse endogenous and exogenous factors have been shown to affect leaf senescence *via* distinct gene networks (Zhang and Zhou, 2013). To identify specific molecular markers for each senescence condition, we identified DEGs that were specifically responsive to age-, dark-, and salt-induced senescence. Three criteria were employed in the selection of these DEGs: 1) gene expression levels (FPKM ≥ 1), 2) significant responsiveness to each senescence condition (i.e., Adjusted-P ≤ 0.05 & |log_2_(FC)| ≥ 1), and 3) senescence responsiveness of homologous Arabidopsis genes. Twenty up- and 20 down-regulated DEGs were selected as specifically responsive DEGs (sDEGs) for each condition (**Figure 5** and **Supplementary Dataset 5**). Of these, the expression profiles of one up-regulated and one down-regulated sDEG for each condition (i.e., UP: *Zj_G01458* and DOWN: *Zj_G12647* for AGE; UP: *Zj_G10221* and DOWN: *Zj_G26206* for DARK; UP: *Zj_G05196* and DOWN: *Zj_G31160* for SALT) were then validated by qRT-PCR using independent biological samples separate from those used for RNA-seq analysis. The expression levels of these six sDEGs were found to be consistent with the results from RNA-seq analysis—that is, they displayed an increase or decrease in expression for the designated senescence condition (**Figure 6**). However, it should be noted that some of these genes, including *Zj_G12647*, *Zj_G10221*, and *Zj_G05196* displayed opposite responsiveness in other senescence conditions (**Figures 6B, C, E**). These findings suggest that the sDEG genes can be used as markers to distinguish between different senescence-inducing conditions. Furthermore, these results also support the validity of the transcriptome sequencing data generated by this study.

### Identification and expression profiles of TF cDEGs

**Figure 7:**
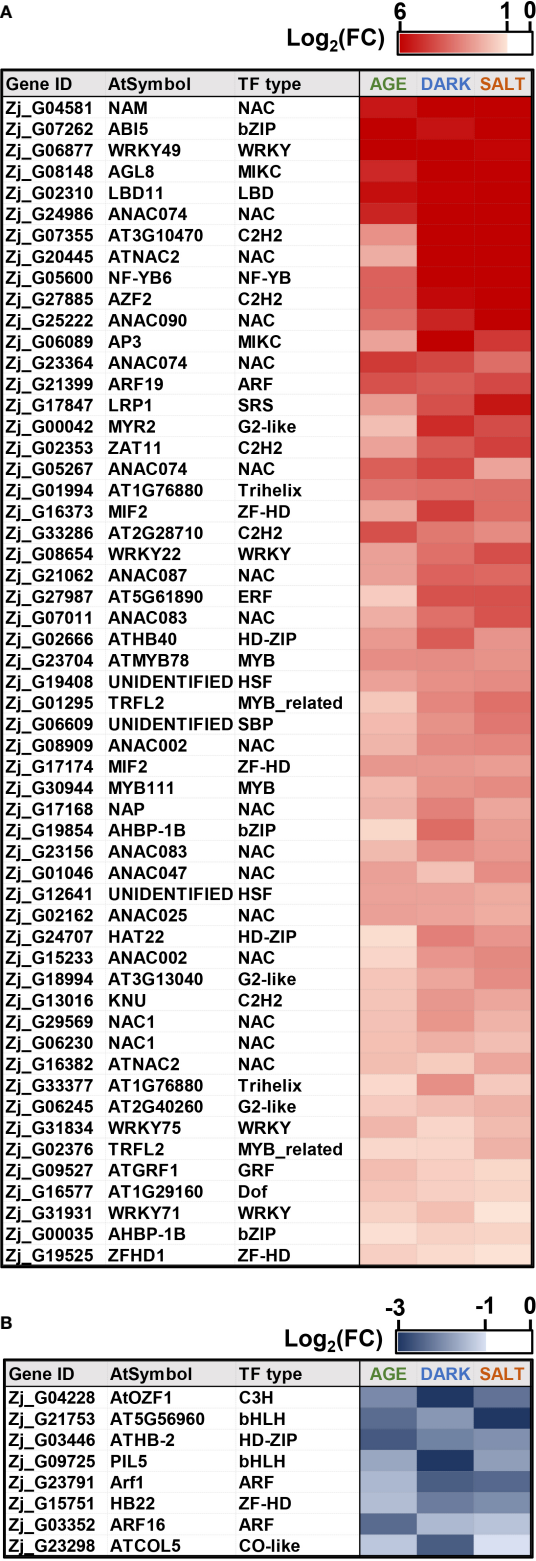
Expression of *Z. japonica* TFs in response to all senescence-inducing conditions. Shown are expression heatmaps of TFs associated with up-regulated **(A)** and down-regulated **(B)** expression under all senescence conditions. Heatmaps use color intensity to indicate |log_2_(FC)| in TF expression for each condition.

**Figure 8:**
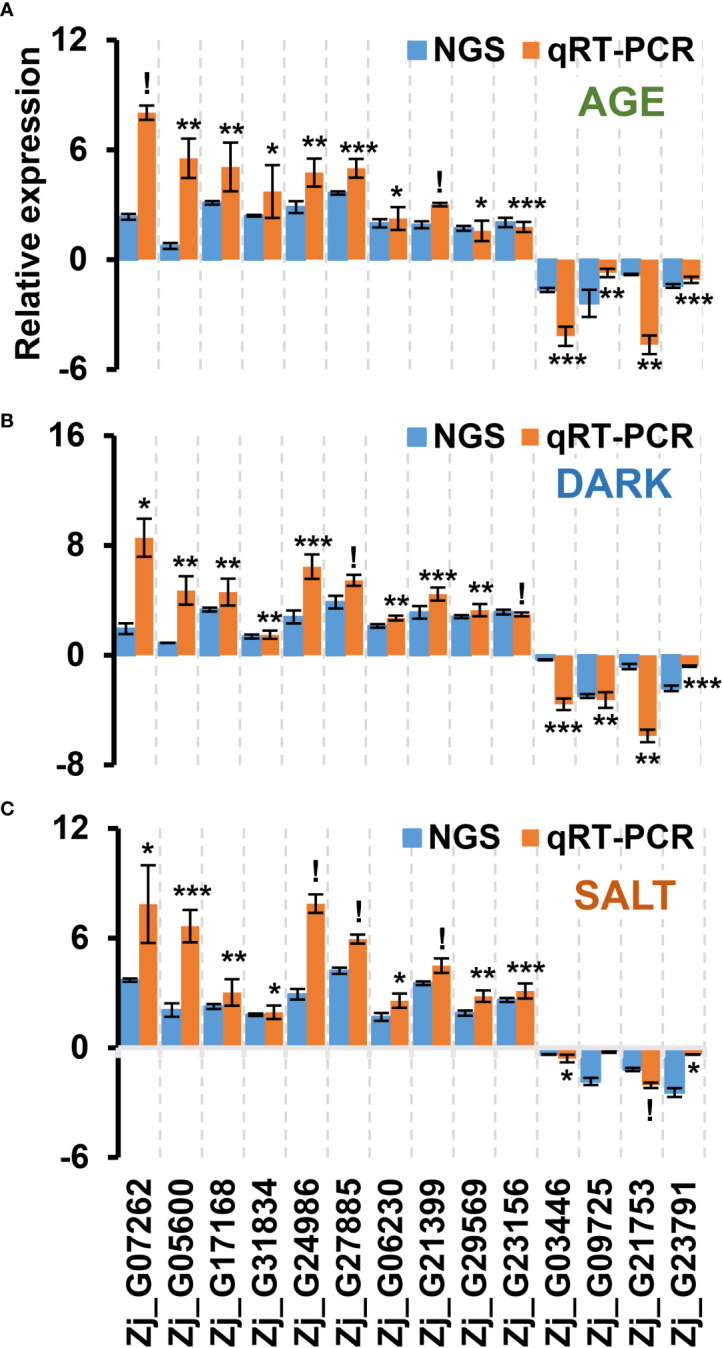
qRT-PCR-based validation of TF DEGs that were responsive to all senescence conditions examined using RNA-seq analysis. Shown are the expression profiles of DEGs identified as commonly responsive to age- **(A)**, dark- **(B)**, and salt- **(C)** induced senescence conditions *via* RNA-seq analysis. The samples used in this qRT-PCR validation were independently prepared from those used in the RNA-seq analyses. Data represent mean ± SE (n = 2 or 4). Statistical analyses were performed using one-way ANOVA test (*p < 0.05; **p < 0.01; ***p < 0.001; and ^!^p < 0.0001).

TFs play a crucial role in regulating leaf senescence, which makes intuitive sense, since senescence is a process that involves changes in the expression of thousands of genes (Bengoa Luoni et al., 2019). Previous studies have also shown that TFs can be used as potential gene resources for altering senescence phenotypes in crops (Sekhon et al., 2012; Jan et al., 2013; Bengoa Luoni et al., 2019; Wang et al., 2021). To identify genes likely to affect senescence, we selected a total of 55 up-regulated TFs and 8 down-regulated TFs that were highly responsive to all senescence-inducing conditions from our list of cDEGs. The selected TFs belonged to representative TF families, including NAC, WRKY, bHLH, and ARF (**Figure 7** and **Supplementary Dataset 6**), all of which have been reported to be associated with senescence regulation (Ahmad and Guo, 2019; Bengoa Luoni et al., 2019). Most of the genes from the bHLH and ARF TF families were found to be down-regulated, while most of the genes from the NAC and WRKY TF families were up-regulated. To confirm the expression profiles of the TF cDEGs, we selected 10 up- and 4 down-regulated genes based on their senescence responsiveness and evaluated their expression profiles under age-, dark-, and salt-induced senescence conditions (**Figure 8**). Of these genes, 13 of 14 (93%) genes were confirmed as cDEGs by qRT-PCR analysis. These include ten up-regulated cDEGs: *Zj_G07262, Zj_G05600, Zj_G17168, Zj_G31834, Zj_G24986, Zj_G27885, Zj_G06230, Zj_G21399, Zj_G29569*, and *Zj_G23156*, and three down-regulated cDEGs: *Zj_G03446, Zj_G21753*, and *Zj_G23791*. It should be noted that *Zj_G09725* only showed transcriptional differences in response to age- and dark-induced senescence.

**Figure 9:**
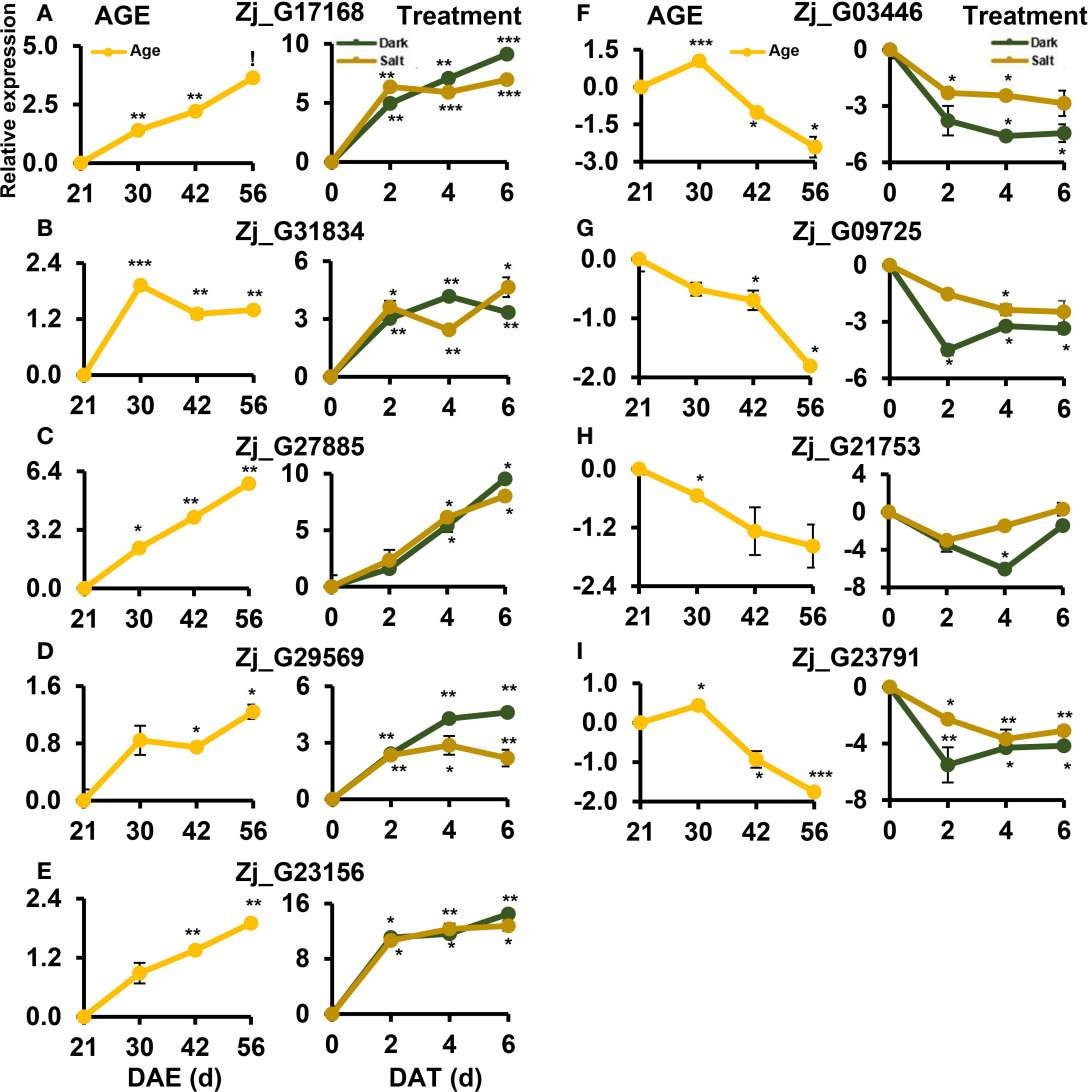
Kinetic expression patterns of TF DEGs identified by RNA-seq analysis along senescence. **(A–E)** Expression of up-regulated DEGs. **(F–I)** Expression of down-regulated DEGs. Samples were prepared as in **Figure 1** but were harvested at the indicated age or treatment. Data represent mean ± SE (n = 2) and are presented as the log_2_(FC) calculated between each condition and their respective controls. Statistical analyses were performed using one-way ANOVA test (*p < 0.05; **p < 0.01; ***p < 0.001; and ^!^p < 0.0001).

Of the TF cDEGs, we selected five up-regulated and three down-regulated genes for further kinetic analysis since their homologous genes are reported to be involved in the regulation of leaf senescence (Li et al., 2020). Besides, *Zj_G09725* was also included in the kinetic analysis as its expression levels may change at other time points. The kinetic expression analysis unveiled diverse expression patterns for these genes across all three conditions with two major patterns of expressions standing out: one that gradually increased throughout the senescence period, and another that peaked early on and maintained that level (**Figure 9**). *Zj_G17168*, *Zj_G27885*, and *Zj_G09725* belonged to the former category, while *Zj_G31834* and *Zj_G29569* were grouped in the latter. However, expression of *Zj_G21753* did not significantly respond to the salt-induced senescence condition (**Figure 9H**). Although these genes exhibited different expression patterns, they are, except for *Zj_G21753*, likely to play a convergent role in senescence regulation. Overall, these findings validate the result of our previous RNA-seq analysis and identify potential regulatory and convergent TF genes that may be involved in the senescence process.

### Effect of TF candidate genes on *Z. japonica* senescence promoters in a protoplast-mediated transient expression system

**Figure 10:**
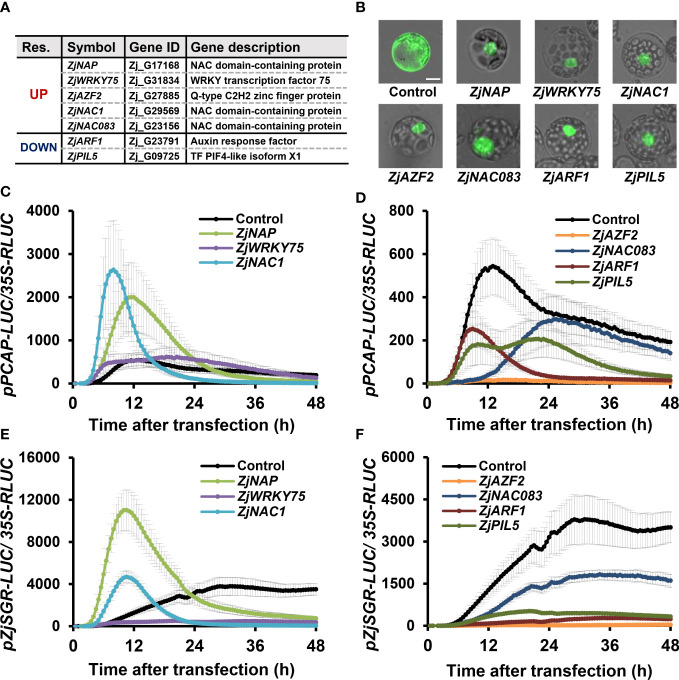
Functional analysis of putative TF candidate genes *via* effector and reporter assays in Arabidopsis protoplasts. **(A)** Information regarding putative TF candidate genes. Res, Response. **(B)** Expression and nuclear localization of proteins encoded by candidate genes in Arabidopsis protoplasts. The indicated genes fused with GFP or GFP control were transiently expressed in Arabidopsis protoplasts. GFP signals were captured with a fluorescence microscope after 24 h of transfection. Bars = 100 µm. **(C–F)** Bioluminescence traces of *ZjPCAP*-LUC **(C, D)** and *ZjSGR-*LUC **(E, F)** in Arabidopsis protoplasts. Effectors were grouped and presented, based on the effectiveness on the *ZjPCAP-*LUC reporter **(C, E)**, Up;**(D, F),** Down). Arabidopsis protoplasts were co-transfected with overexpression effectors of GFP (control) or with the indicated gene fused with GFP and either a reporter of *ZjPCAP*- or *ZjSGR-*LUC. Luciferase activity was normalized to the maximum level of 35S:RLUC. Data represent mean ± SE (n = 6).

Transcriptomic analysis and further qRT-PCR experiments revealed that five up-regulated and three down-regulated TFs were highly responsive to all senescence conditions in *Z. japonica*, suggesting they may play key roles in regulating leaf senescence. To evaluate the regulatory functions of these candidates in leaf senescence, we performed a protoplast-based senescence assay using senescence promoter-driven LUC reporters (Doan et al., 2022). Since these candidate genes are members of known TF families (**Figure 10A**), the proper expression of TF protein-tagged eGFP was confirmed by their nuclear localization in Arabidopsis protoplasts (**Figure 10B**).

A protoplast-based senescence assay was performed using the *Z. japonica* promoters for *ZjPCAP* and *ZjSGR* as reporter promoters; these genes respond during the early and late stages of senescence progression, respectively. Luciferase reporters driven by *ZjPCAP* and *ZjSGR* (i.e., *ZjPCAP-LUC* and *ZjSGR-LUC*) were co-transfected with GFP-fused TF effector plasmids and a GFP-only control into Arabidopsis protoplasts. The time series of luminescence levels were then examined and compared (**Figures 10C–F**). The expression levels of *ZjPCAP-LUC* and *ZjSGR-LUC*, when co-transfected with the GFP control, were significantly induced, but they showed different patterns. *ZjPCAP-LUC* exhibited an earlier peak followed by a gradual reduction, while *ZjSGR-LUC* showed a steady level with a later peak. When *ZjNAP-GFP* and *ZjNAC1-GFP* were expressed as effectors, *ZjPCAP-LUC* and *ZjSGR-LUC* were induced earlier and reached their peak levels earlier than the GFP control alone did (**Figures 10C, E** and **Supplementary Figures 1A, C**). This indicates that *ZjNAP* and *ZjNAC1* may function as potential positive senescence regulators. In contrast, *ZjAZF2*, *ZjNAC083*, *ZjARF1*, and *ZjPIL5* were found to strongly suppress the expression of *ZjPCAP-LUC* and *ZjSGR-LUC*, indicating that they may play roles as negative senescence regulators (**Figures 10D, F** and **Supplementary Figures 1B, D**). However, the expression of two reporter genes when *ZjHB2* was expressed was comparable to that when the GFP control was expressed, indicating that *ZjHB2* is not likely to be a senescence regulator (**Supplementary Figure 2**). Interestingly, *ZjWRKY75* caused earlier induction of *ZjPCAP-LUC* and earlier reduction of *ZjSGR-LUC* than the control (**Figures 10C, E** and **Supplementary Figures 1A, C**). *ZjWRKY75* may play a dual role during senescence by positively regulating senescence during early stages and by playing a negative and/or attenuating role during late stages. Overall, these seven TFs cDEGs were found to be transcriptionally responsive to a broad range of senescence-inducing conditions and may therefore be involved in senescence regulation in *Z. japonica*.

## Discussion

*Z. japonica* is a widely cultivated warm-season turfgrass with high economic value. It is commonly employed as a decorative cover in diverse settings, including gardens, parks, sport fields, and golf courses. However, *Z. japonica*, despite its popularity, exhibits an early yellowing phenotype in the fall (Zhang et al., 2022). The shortness of its green period can be mitigated through controlling senescence (Guan et al., 2022a), and therefore delaying leaf senescence may be an effective means of extending the green phase of *Z. japonica* and thereby enhancing its economic value.

Leaf senescence represents the ultimate stage of leaf development and is regulated by complex processes. Although leaf senescence is a common and acquired developmental event, senescence responses vary among species (Panchen et al., 2015; Kim et al., 2016b; Chai et al., 2019). Senescence is considered a significant economic trait in many crops, including *Z. japonica*; however, the molecular mechanisms underlying senescence in *Z. japonica* remain largely unknown. The onset of senescence is primarily regulated by leaf age but can also be affected by external stresses such as salt treatment and limited energy levels, as seen following dark treatment. Although age, dark, and salt can induce leaf senescence with yellowing, their physiological and molecular responses during senescence are reported to be diverse (Allu et al., 2014; Lyu et al., 2017; Kim et al., 2018c; Lyu et al., 2019). In this study, we conducted comparative transcriptomic analysis of *Z. japonica* subjected to age-, dark-, and salt-induced senescence conditions to obtain a comprehensive understanding of the molecular mechanisms underlying *Z. japonica* leaf senescence to identify senescence regulatory genes that could potentially be used for genetic modification of *Z. japonica* to delay leaf senescence.

Chlorophyll breakdown is a widely recognized physiological marker of leaf senescence responses and is triggered by various factors, including age, stress, hormones, and dark (Song et al., 2014). By monitoring leaf yellowing in conjunction with taking chlorophyll content measurements, the progression of senescence can be evaluated and compared across different senescence-inducing conditions. Since the molecular responses that are important during early senescence differ among various senescence conditions, comparative transcriptome analyses should be conducted at similar and early stages of senescence (Guo and Gan, 2012). Furthermore, the early senescence stage is considered useful for identifying regulatory genes that can be used for genetic modification to regulate leaf senescence (Lim et al., 2007). Here, as previously employed by other studies, a 20% chlorophyll loss in senescing leaves was used as an indicator of the end of the early senescence stage (**Figures 1A–D**) (Woo et al., 2010; Kim et al., 2018a).

Transcriptomic analyses have been highly successful in elucidating the molecular mechanisms underlying leaf senescence in Arabidopsis (Lin and Wu, 2004; Buchanan-Wollaston et al., 2005; Breeze et al., 2011; Woo et al., 2016; Kim et al., 2018c). The advent of next generation sequencing-based RNA-seq protocols has greatly enhanced our ability to identify senescence-associated RNAs, including novel transcripts, small RNAs, and long noncoding RNAs (Woo et al., 2016; Kim et al., 2022). RNA-seq has also been widely used to discover and identify genes implicated in senescence in crop species (Kong et al., 2013), particularly in non-model organisms where a reference genome is not available. While genome sequence resources for *Z. japonica* were published in 2016 (Tanaka et al., 2016), the gene annotation of the reference genome remains primitive and has not been updated in the National Center for Biotechnology Information database. This lack of comprehensive genome annotation has hindered the generation of genetic resources for *Z. japonica*. However, recent long-read sequencing data for *Z. japonica* has permitted exploration of a full-length transcriptome and has improved transcript annotation for its reference genome (Guan et al., 2022b). In addition, continuous refinement and routine annotation updates are required for correctly interpreting functional elements of the genome. Temporal and spatial RNA-seq analyses—including analyses of leaf senescence, as in this study—can improve the accuracy of the genome information for *Z. japonica* (**Supplementary Dataset 1** and **Supplementary File 1**).

Functional classification of the transcriptomic responses of *Z. japonica* during age-, dark-, and salt-induced senescence conditions can help to explain the underlying biological processes involved in leaf senescence (**Figure 3**). These different senescence conditions share key processes, including the up-regulation of amino acid degradation and the down-regulation of photosynthesis. Of the up-regulated processes specific to individual senescence conditions, programmed cell death, cell wall reorganization, and solute transport were identified as representative biological processes specifically enriched in the age-, dark-, and salt-induced senescence conditions, respectively. Conversely, cytoskeleton reorganization, glycolysis, and TCP TF expression were found to be specifically down-regulated by age-, dark-, and salt-induced senescence conditions, respectively. These results are consistent with previous transcriptomic analyses of Arabidopsis leaf senescence (Breeze et al., 2011; Allu et al., 2014; Woo et al., 2016; Kim et al., 2018c). Notably, chromatin organization and gibberellin action were also found to be up- and down-regulated processes, respectively, in age-induced leaf senescence; to our knowledge, this finding has not yet been reported in another study. These findings may therefore be specifically related to leaf senescence in *Z. japonica*.

Our transcriptomic analyses also identified a large number of sDEGs and cDEGs in various senescence-induced conditions (**Figures 4**, **5**, **7**). Several of these were validated as DEGs in biological samples that were independent of those used in the RNA-seq analysis (**Figures 6**, **8**). Despite several marker genes—including *ZjSGR*, *ZjSAG14*, and *ZjNOL*—being identified as general senescence markers by previous studies (Teng et al., 2016a; Dong et al., 2022a; Guan et al., 2022a), a comprehensive analysis of markers specific to responses to age-, dark-, and salt-induced senescence has been limited. Here, we identified and validated six sDEGs and fourteen cDEGs that can be used as markers for dissecting senescence-triggering factors and exploring the progression of senescence responses, respectively.

TFs have been demonstrated to play crucial roles as hubs in the senescence regulatory network, since they regulate the transcription of many downstream genes (Balazadeh et al., 2008). Our analysis identified 55 up-regulated and 8 down-regulated TF cDEGs, the majority of which belonged to the NAC, WRKY, bHLH, and ARF families (**Figure 7**). NAC TFs have been found to be involved in chlorophyll breakdown and leaf senescence, and are actively implicated in abscisic acid, methyl jasmonate, and ethylene signaling, which can counteract leaf senescence (Balazadeh et al., 2008; Takasaki et al., 2015). The ARF TF family plays a major role in responses to abiotic and biotic stress, and in defense, while the bHLH, MYB, and WRKY families have been primarily implicated in mediating senescence (Tolosa and Zhang, 2020; Dong et al., 2022b). It is certain that the TF DEGs identified in this study also play important roles in regulating senescence by mediating various senescence-related biological processes in *Z. japonica*.

While many transcriptomic studies have identified TFs associated with leaf senescence in various non-model plants, their functional roles remain largely unknown (Woo et al., 2013; Fraga et al., 2021; Zhang et al., 2021). This is because functional studies that use knock-out or transgenic approaches can be time- and resource-consuming, especially in crop species (Mahmood et al., 2022). In *Z. japonica*, functional analyses of SAGs have been performed by transgenic approaches that introduce them in Arabidopsis. Arabidopsis plants expressing *ZjSGR, ZjPPH*, and *ZjNOL* genes that involved in chlorophyll degradation, exhibited rapid chlorophyll degradation and senescence; this phenotype closely resembled functional studies of homologous genes in Arabidopsis (Teng et al., 2016b; Teng et al., 2021; Guan et al., 2022a). Taken together, these findings suggest that SAGs have a conserved regulatory role in Arabidopsis and *Z. japonica*, although the number of genes tested is limited. We employed a cell-based senescence assay using transient expression in Arabidopsis protoplasts to rapidly evaluate the function of candidate senescence-related genes. The protoplast transient expression system provides a platform for rapid and high-throughput assays using promoter-driven LUC reporters (Hwang and Sheen, 2001; Yoo et al., 2007; Huo et al., 2017). We selected *ZjSGR* and *ZjPCAP* promoters as senescence reporter promoters, since they showed different but strong induction patterns during senescence (**Figures 10C–F**). Based on their expression patterns, five up-regulated and two down-regulated TF DEGs—representing members of the NAC, WRKY, C2H2, ARF, and bHLH families—were selected for functional assays in protoplasts (**Figure 9**). Our protoplast-based senescence assay revealed that *ZjNAP* and *ZjNAC1* function as positive senescence regulators, while *ZjNAC083, ZjAZF2, ZjARF1*, and *ZjPIL5* function as negative senescence regulators (**Figure 10** and **Supplementary Figure 1**). Notably, *ZjAZF2* and *ZjNAC083* were identified as up-regulated DEGs, but function as negative senescence regulators, suggesting that they may act as molecular brakes that slow down the senescence process. Alternatively, they may act as molecular breakers that shut down the inappropriate onset of senescence during aging, similar to the NAC troika (Kim et al., 2018a). Our functional assessment based on rapid senescence assays using the protoplast transient expression system supports the possibility that these seven TFs likely function as senescence regulatory genes in *Z. japonica*. To date senescence regulatory genes identified in *Z. japonica* are involved in the chlorophyll degradation processes (Teng et al., 2016b; Teng et al., 2021; Guan et al., 2022a). In-depth studies of all DEGs, including seven TFs tested here, is an important topic for future research, since this may improve our understanding of transcriptional regulation during leaf senescence in *Z. japonica*. Furthermore, gene editing and overexpression approaches can be applied to study whether these putative positive and negative senescence regulators delay leaf senescence, since this may lead to the generation of the desired evergreen trait in this species.

In summary, our comparative transcriptome analysis of age-, dark-, and salt-induced *Z. japonica* senescence has provided a comprehensive molecular understanding of key transcriptional events during leaf senescence in this species. This study has also identified potential genetic resources for breeding *Z. japonica* with a prolonged green phase. The results of this study therefore offer a deeper understanding of the molecular mechanisms underlying leaf senescence in *Z. japonica* and provide valuable information for the development of evergreen cultivars of this species.

## Data availability statement

The original contributions presented in the study are publicly available. This data can be found here: NCBI BioProject, accession PRJNA934408.

## Author contributions

LW, PPTD, H-YL, JHK, and JK conceived and designed the experiments. LW, PPTD, NNC, and JHK performed the experiments. LW, PPTD, and JK analyzed the data. LW, PPTD, JHK, and JK wrote the paper. All authors contributed to the article and approved the submitted version.

## Supplementary material

Supplementary Dataset 1 Gene information of new annotated gene ID and their comparison to old *Z. japonica* gene ID.

Supplementary Dataset 2 Quantile nomalized values of Log_2-_based FPKM in each condition.

Supplementary Dataset 3 Z-score enrichment of MapMan categories in transcriptome profiles under age-, dark-, and salt-induced senescence.

Supplementary Dataset 4 Selection of DEGs in each condition.

Supplementary Dataset 5 Expression levels of top 20 up and down-regulated cDEGs.

Supplementary Dataset 6 Expression levels of TF cDEGs.

Supplementary File 1 GTF file of new annotated *Z. japonica* gene ID used in this study.
