# Supplementary material for: Comprehensive transcriptomic analysis of age-, dark-, and salt-induced senescence reveals underlying mechanisms and key regulators of leaf senescence in Zoysia japonica
Source: Front Plant Sci. 2023 May 30;14:1170808. doi: 10.3389/fpls.2023.1170808 (PMC10265201; doi:10.3389/fpls.2023.1170808)
Supplement: Supplementary Dataset 1 — Gene information of new annotated gene ID and their comparison to old Z. japonica gene ID. [file DataSheet_1.zip › Supplementary information.docx]

SUPPLEMENTARY INFORMATION

Comprehensive transcriptomic analysis of age-, dark-, and salt-induced senescence reveals underlying mechanisms and key regulators of leaf senescence in *Zoysia japonica*

Lanshuo Wang^1, !^, Phan Phuong Thao Doan^1, !^, Nguyen Nguyen Chuong^1^, Hyo-Yeon Lee^2,3^, Jin Hee Kim ^2,*^ and Jeongsik Kim ^1,2,4,*^

^1^Interdisciplinary Graduate Program in Advanced Convergence Technology & Science, Jeju National University, Jeju 63243, South Korea

^2^Subtropical Horticulture Research Institute, Jeju National University, Jeju 63243, South Korea

^3^Department of Biotechnology, Jeju National University, Jeju 63243, Republic of Korea

^4^Faculty of Science Education, Jeju National University, Jeju 63243, South Korea

^!^ These authors contributed equally to this work.

*** Correspondence:**jinheekim@jejunu.ac.kr (J.H.K); yorus@jejunu.ac.kr (J.K.)

**This file includes:**

Supplementary Figures 1 and 2

Supplementary Tables 1, 2, 3, and 4

**Other supplementary information for this manuscript includes the following:**

Supplementary Dataset 1, 2, 3, 4, 5, and 6

Supplementary File 1_New annotated Z. japonica gene ID.gtf

# Supplementary Figures and Tables

## Supplementary Figure


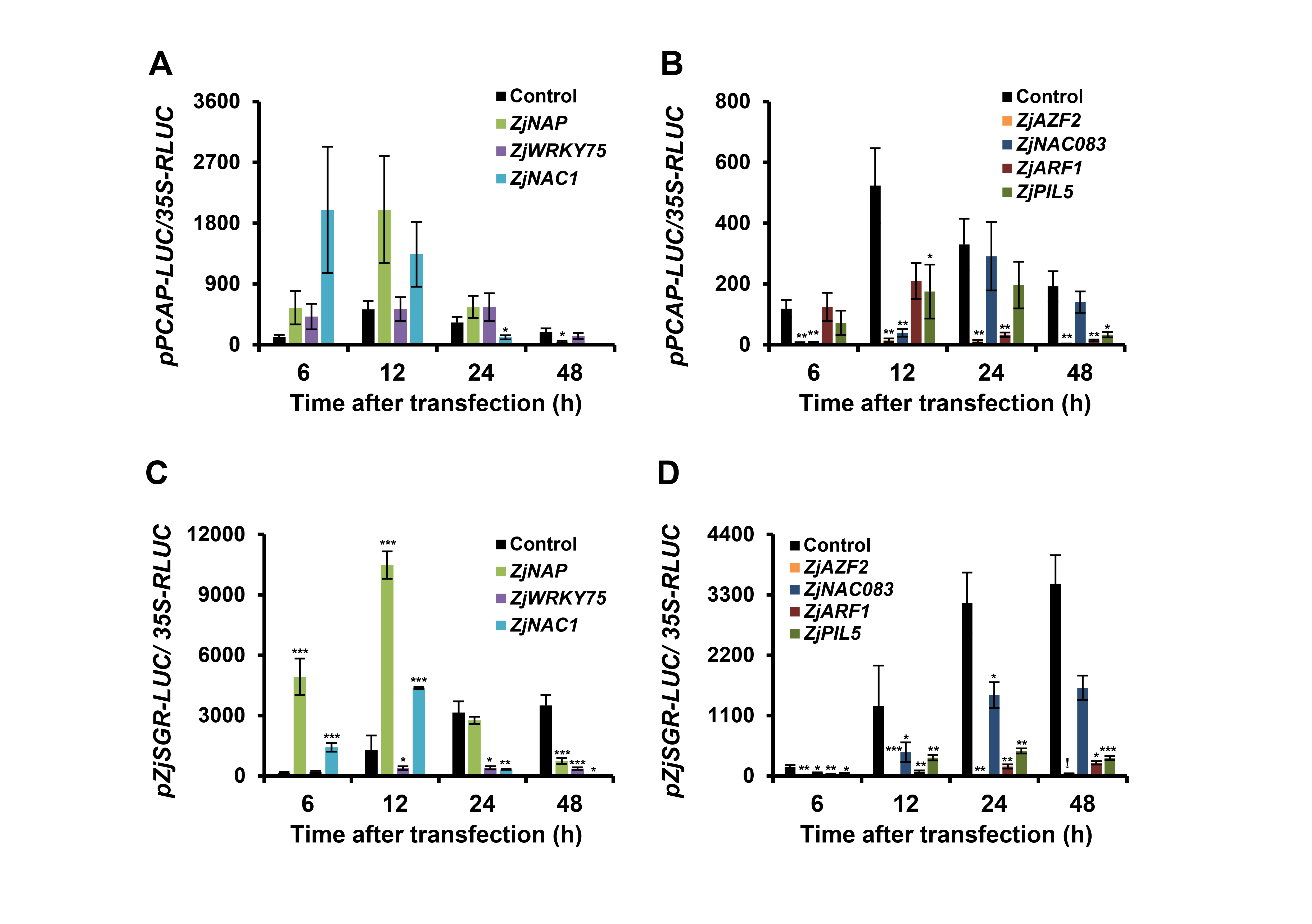


**Supplementary Figure 1. Functional analysis of putative TF candidate genes through effector and reporter assay in Arabidopsis protoplasts. (A-D)** Normalized expression of *ZjPCAP*-LUC (**A, B**) and *ZjSGR-*LUC (**C**, **D**) in Arabidopsis protoplasts. Effectors were grouped and presented, based on the effectiveness on the *ZjPCAP-*LUC reporter (**A** and **C**, Up; **B** and **D**, Down). Data represent mean ± SE (n = 6). Statistical analyses were performed using one-way ANOVA test (*p < 0.05; **p < 0.01; ***p < 0.001; and ^!^p < 0.0001).


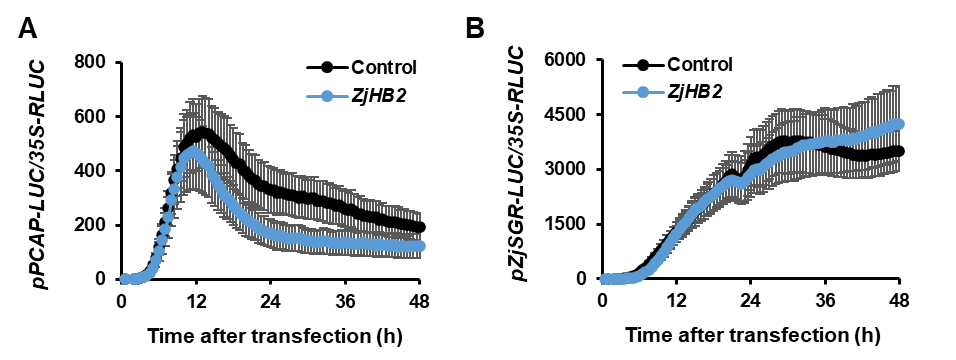


**Supplementary Figure 2. No effect of *ZjHB2* in the expression of *ZjPCAP-LUC* and *ZjSGR-LUC* in Arabidopsis protoplasts.**

Bioluminescence traces of *ZjPCAP*-LUC (**A**) and *ZjSGR-*LUC (**B**) in Arabidopsis protoplasts. Protoplast transfection and luminescence measurement was performed as in Figure 10 except for using GFP-ZjHB2 as an effector. Data represent mean ± SE (n = 6).

## Supplementary tables

**Supplementary Table 1. List of gene-specific PCR primer sequences for cloning**

| **Primer Name** | **Sequence (5’ to 3’)** | **R.E.*** |
| --- | --- | --- |
| *ZjNAP*-F | TTTACTAGTATGGCGACGAGGATGCCTT | *Spe*I |
| *ZjNAP*-R | TTTAAGCTTCTGGTTCAGGAACGGGTGGCTA | *Hind*III |
| *ZjWRKY75*-F | TTTCTGCAGATGGAGAGCAACTACCATCC | *Pst*I |
| *ZjWRKY75*-R | TTTAGGCCTCCGGAACATTGGGCTACT | *Stu*I |
| *ZjAZF2*-F | TTTCTGCAGATGGCGGTAGACGCGATCAT | *Pst*I |
| *ZjAZF2*-R | TTTAGGCCTGGCCGGGATCATGAGCCG | *Stu*I |
| *ZjNAC1*-F | TTTCTGCAGATGTCGATGAGTTTCGTGAG | *Pst*I |
| *ZjNAC1*-R | TTTAAGCTTGAAGTGATTCATCCATGTAG | *Hind*III |
| *ZjNAC083*-F | TTTACTAGTATGGACGCGAAGGAGGTGGT | *Spe*I |
| *ZjNAC083*-R | TTTAGGCCTCGCGCAGCCTCCGCTGGTGGTGT | *Stu*I |
| *ZjARF1*-F | TTTCTGCAGATGGCCGCGCCGATGGAGGTGT | *Pst*I |
| *ZjARF1*-R | TTTAGGCCTATCAGATGGTGAGTTTACAG | *Stu*I |
| *ZjPIL5*-F | TTTCTGCAGATGGATGGTAAGGCGAGGTC | *Pst*I |
| *ZjPIL5*-R | TTTAGGCCTAACTCCATTAGTAGGTGGCA | *Stu*I |
| *ZjHB2-F* | TTTACTAGTATGATGGAGAGGGCAGATGA | *SpeI* |
| *ZjHB2-R* | TTTAGGCCTGCTGCTGGCAAGCGACTGCA | *StuI* |
| *ZjPCAP1*-F | TTTGGACTTCATGTATTGCATATCGTTTA | *Bam*HI |
| *ZjPCAP1*-R | TTTAGGCCTGGCTGGAAACGAGGCCAAAT | *Stu*I |

* Restriction enzyme

**Supplementary Table 2. List of PCR primers of genes that used for qRT-PCR**

| **Gene ID** | **Forward Sequence (5’ to 3’)** | **Reverse Sequence (5’ to 3’)** | **DEG Type** |
| --- | --- | --- | --- |
| *ZjSGR* | CGTCCACTGCCACATCTCCG | CGAACGCCTTCAGCACCACA | N/A |
| *COR410* | GAAGCACGGGTCAAGAAGG | TCTCGTCGATCACCTCCTCT | N/A |
| *ZjDREB1* | TTGGAGGCTGCTCATGCATA | TTCAACGCATGCACCTCAGT | N/A |
| *ZjACT* | GGTCCTCTTCCAGCCATCCTTC | GTGCAAGGGCAGTGATCTCCTTG | N/A |
| *Zj_G08090* | AGAGATCACGCAAGAAATCCAT | CTGCGCCACTAATAAGATGTTG | Specific |
| *Zj_G12647* | GGAATTCAGCAAGTCAAACTCC | TCGATCTCTGGTCTTTTTCCTC | Specific |
| *Zj_G10221* | CTGGTATTGAGCCTTACCTTGG | ACAGATTTTGCACGACAATCAC | Specific |
| *Zj_G26206* | TATTTGCATGAGAGGTTGATCG | CAACTCTGCGATGTTCTCTACG | Specific |
| *Zj_G05196* | TATCATCAGGGCGAAGCTTATT | CAGGAGGTAAAACAGCCAAGTC | Specific |
| *Zj_G31160* | GAATTCACGAACTCTGCCTCTT | AGCACTGGATCCATCATTTTCT | Specific |
| *Zj_G07262* | AAAGAGAGAGATGCTGGTGGAG | CATATTGGAACGACACAATGCT | Common |
| *Zj_G05600* | TTCATGTGAGAGTTGGACCTGT | TACATATATTGGGACGGCATGA | Common |
| *Zj_G17168* | ACGTTTCGCTGGTTGTATCTTT | CTCAGCAACTGGATAAGGGTTC | Common |
| *Zj_G02756* | TGCTTCAGGGGAGCTACTACAT | TCCCAAACAAACAGAATCACAC | Common |
| *Zj_G31834* | CTGTTGCTTGTCCAACTCTACG | TGTAGAGGGGTTAATGGATGCT | Common |
| *Zj_G24986* | TTGGGTTGGTCATTAGACTGTG | ACAGGCACCACTTCTCATTTCT | Common |
| *Zj_G27885* | TTAGTCGTGTTGGATTTGCTTG | TCGACTGAACCAATCAATGAAG | Common |
| *Zj_G04249* | CCTGTACGGCACTTAGGACTTC | TACGTGCGACATACTGCTTTCT | Common |
| *Zj_G06230* | GAATTCCGTATGGAAGGACAAG | TGTGTCAACATTGTAGCTGCTG | Common |
| *Zj_G21399* | AATCAGGAGGGCTAACAGACAA | ATGGTAAATGGGCTATTGTTGG | Common |
| *Zj_G13346* | CAGGCCTATTGCTACTCTGCTT | TTCCGATCCAAAATAACTTGCT | Common |
| *Zj_G29569* | CTTGAGGTAGACCCGCCTATAA | GACTAGTCAGTCGTGCTTGCAT | Common |
| *Zj_G23156* | AATTAGCATCGCAAGAGAGAGC | TGGACAAGGATCAGGTAGCAAT | Common |
| *Zj_G03446* | GAGCTTGGCTTTGATTAGCAGT | ACCATATCCAGCTGCTTTTCTC | Common |
| *Zj_G09725* | CAATGATGTTTCCAGGTGCTTA | ATTTGACCTAGGCTTTGAGCTG | Common |
| *Zj_G21753* | GAGGCGAAGACTTGAGACAACT | GGGACCGAAGGAGTGATAAATA | Common |
| *Zj_G23791* | CTCCGTAGACTTTGGCTCAGAT | TTGCATAATTACCTTGGTGCAG | Common |
| *Zj_G07719* | CAGGAGTGCTACAAAGGCTTCT | TTCTCGTTCCACCTCTCTAAGG | Common |

**Supplementary Table 3. QC reports for raw sequencing reads**

| **Samples** | **Total read base** | **Total reads** | **QC(%)** | **Q30(%)** |
| --- | --- | --- | --- | --- |
| Control-light-1 | 9,007,616,926 | 89,184,326 | 53.3 | 96.6 |
| Control-light-2 | 8,018,001,150 | 79,386,150 | 52.8 | 96.4 |
| Control-light-3 | 9,013,979,724 | 89,247,324 | 53.0 | 96.6 |
| Salt-1 | 8,470,380,554 | 83,865,154 | 51.0 | 96.3 |
| Salt-2 | 7,692,189,694 | 76,160,294 | 51.8 | 96.6 |
| Salt-3 | 8,574,612,150 | 84,897,150 | 52.4 | 96.5 |
| Dark-1 | 8,814,549,770 | 87,272,770 | 52.2 | 96.5 |
| Dark-2 | 8,795,829,824 | 87,087,424 | 52.5 | 96.5 |
| Dark-3 | 8,370,741,428 | 82,878,628 | 52.3 | 96.5 |
| MG-1 | 7,220,428,794 | 71,489,394 | 53.0 | 96.5 |
| MG-2 | 7,579,553,080 | 75,045,080 | 53.3 | 96.6 |
| MG-3 | 7,418,014,892 | 73,445,692 | 53.4 | 96.6 |
| SS-1 | 7,535,835,028 | 74,612,228 | 52.2 | 96.2 |
| SS-2 | 9,036,141,750 | 89,466,750 | 53.1 | 96.6 |
| SS-3 | 8,987,696,090 | 88,987,090 | 53.2 | 96.6 |

· Total read bases = Total reads x Read length

· Total read bases: Total number of bases sequenced

· Total reads: Total number of reads

· GC (%): GC content

· Q30 (%): Ratio of bases that have phred quality score greater than or equal to 30

**Supplementary Table 4. Statistics of mapping results using *De novo* mapping-based genome structure annoation**

| **Samples** | **Total  transcripts No.** | **Transcript  matching ratio(%)** | **Transcript No. with new annotation file** | **Transcript matching ratio with new annotation file(%)** |
| --- | --- | --- | --- | --- |
| Control-light-1 | 35,662 | 76.3 | 33,878 | 94.1 |
| Control-light-2 | 35,612 | 76.7 | 33,831 | 94.0 |
| Control-light-3 | 35,936 | 74.5 | 34,139 | 94.9 |
| Salt-1 | 36,050 | 73.6 | 34,247 | 95.2 |
| Salt-2 | 35,693 | 73.9 | 33,908 | 94.2 |
| Salt-3 | 34,982 | 73.8 | 33,181 | 92.2 |
| Dark-1 | 36,136 | 75.1 | 34,329 | 95.4 |
| Dark-2 | 35,902 | 76.4 | 34,106 | 94.8 |
| Dark-3 | 35,332 | 74.3 | 33,565 | 93.3 |
| MG-1 | 35,530 | 80.7 | 33,753 | 93.8 |
| MG-2 | 34,891 | 75.2 | 33,146 | 92.1 |
| MG-3 | 33,645 | 72.5 | 31,962 | 88.8 |
| SS-1 | 35,211 | 79.7 | 33,450 | 93.0 |
| SS-2 | 34,030 | 74.4 | 32,587 | 90.6 |
| SS-3 | 34,195 | 71.5 | 32,485 | 90.3 |

Transcript matching ratio of our samples using available *Z. joponica* genome (Tanaka et al., 2016; 2^nd^ and 3^rd^ columns) and a new genome structure annotation based on *De novo* mapping from our RNA-seq (4^th^ and 5^th^ columns)
